# Cerebrospinal fluid VILIP-1 and YKL-40, candidate biomarkers to diagnose, predict and monitor Alzheimer’s disease in a memory clinic cohort

**DOI:** 10.1186/s13195-015-0142-1

**Published:** 2015-09-17

**Authors:** Maartje I. Kester, Charlotte E. Teunissen, Courtney Sutphen, Elizabeth M. Herries, Jack H. Ladenson, Chengjie Xiong, Philip Scheltens, Wiesje M. van der Flier, John C. Morris, David M. Holtzman, Anne M. Fagan

**Affiliations:** Alzheimer Center and Department of Neurology, VU University Medical Center, PO box 7057, 1007 MB Amsterdam, The Netherlands; Department of Clinical Chemistry, VU University Medical Center, Amsterdam, The Netherlands; The Knight Alzheimer’s Disease Research Center, Washington University School of Medicine, 660 South Euclid, Campus Box 8111, St Louis, 63110 MO USA; Department of Neurology, Washington University School of Medicine, 660 South Euclid, Campus Box 8111, St Louis, 63110 MO USA; Hope Center for Neurological Disorders, Washington University School of Medicine, 660 South Euclid, Campus Box 8111, St Louis, 63110 MO USA; Department of Pathology and Immunology, Washington University School of Medicine, 660 South Euclid, Campus Box 8111, St Louis, 63110 MO USA; Division of Biostatistics, Washington University School of Medicine, 660 South Euclid, Campus Box 8111, St Louis, 63110 MO USA; Department of Epidemiology and Biostatistics, VU University Medical Center, Amsterdam, The Netherlands

## Abstract

**Introduction:**

We examined the utility of cerebrospinal fluid (CSF) proteins, Chitinase-3-like protein 1 (CHI3L1 or YKL-40), a putative marker of inflammation, and Visinin-like protein-1 (VILIP-1), a marker for neuronal injury, for diagnostic classification and monitoring of disease progression in a memory clinic cohort.

**Methods:**

CSF levels of YKL-40 and VILIP-1 were measured in 37 cognitively normal, 61 Mild Cognitive Impairment (MCI) and 65 Alzheimer’s disease (AD) patients from the memory clinic-based Amsterdam Dementia Cohort who underwent two lumbar punctures, with minimum interval of 6 months and a mean(SE) interval of 2.0(0.1) years. Mean(SE) cognitive follow-up was 3.8 (0.2) years. ANOVA was used to compare baseline differences of log-transformed CSF measures. Cox proportional hazard models were used to evaluate disease progression as a function of CSF tertiles. Linear mixed models were used to evaluate longitudinal change over time. All analyses were sex and age adjusted.

**Results:**

Baseline levels of YKL-40, but not VILIP-1, were higher in MCI and AD patients compared to cognitively normal individuals (mean (SE) pg/mL, 304 (16) and 288 (12) vs. 231 (16), *p* = 0.03 and *p* = 0.006). Baseline levels of both YKL-40 and VILIP-1 in MCI predicted progression to AD (HR 95 % CI = 3.0 (1.1–7.9) and 4.4 (1.5–13.0), respectively, for highest vs. lowest tertile). YKL-40 increased longitudinally in patients with MCI and AD (mean (SE) pg/mL per year, 8.9 (3.0) and 7.1 (3.1), respectively), but not in cognitively normal individuals, whereas levels of VILIP-1 increased only in MCI (mean (SE), 10.7 (2.6) pg/mL per year).

**Conclusions:**

CSF levels of YKL-40 may have utility for discriminating between cognitively normal individuals and patients with MCI or AD. Increased levels of both YKL-40 and VILIP-1 may be associated with disease progression. These CSF biomarkers should be considered for future evaluation in the characterization of the natural history of AD.

## Introduction

Major efforts are underway to develop therapeutic strategies to slow the progression of Alzheimer’s disease (AD). To evaluate the effect of these interventions, biological markers are needed that reflect progression of AD pathology. Cerebrospinal fluid (CSF) biomarkers amyloid beta 1–42 (Aβ42), total tau (tau), and tau phosphorylated at threonine 181 (ptau-181) reflect the neuropathology of AD and are useful as diagnostic markers for AD [5]. In addition, these “classical CSF biomarkers” predict progression to AD in cognitively normal individuals and individuals with mild cognitive impairment (MCI) [16, 18, 23, 47]. Several studies have evaluated whether these classical CSF biomarkers could also be used as markers to monitor progression of AD pathology in affected individuals but, to date, their levels have not been shown to be optimal markers for (therapeutic) monitoring in longitudinal studies owing to their relative stability in the later clinical stages of AD [6, 10, 14, 22, 33, 44, 48].

We hypothesized that biomarkers of neuroinflammation and neurodegeneration could be useful for monitoring disease progression in clinical AD, since amyloid plaque deposition and tau tangle formation are early pathologic processes, while neuroinflammation and neurodegeneration may mark events downstream of Aβ and tau pathology [20]. Two relatively novel biomarkers, Chitinase-3-like protein 1 (YKL-40; a secreted 40 kDa glycoprotein, also known as CHI3L1) and Visinin-like protein-1 (VILIP-1), have recently been shown to have additional diagnostic and prognostic value in distinguishing individuals with symptomatic AD from controls [3, 4, 15, 27, 35, 42, 43]. As an astrocyte-derived and/or microglia-derived protein, YKL-40 has been suggested to be associated with neuroinflammation, a process also hypothesized as a major contributor to cognitive decline in AD [7, 8, 15, 30]. VILIP-1 is a neuronal calcium-sensor protein that has previously been reported to be increased in the central nervous system CSF following stroke [12, 24, 40], and thus is considered a marker of neuronal injury.

We aimed to evaluate the performance of these CSF biomarkers for diagnostic and prognostic purposes in AD, and additionally to assess their longitudinal value for the monitoring of disease progression. To achieve this, we evaluated a memory clinic cohort of AD and MCI patients and cognitively normal subjects who had available repeat CSF samples collected over time.

## Methods

### Patients

The cohort was comprised of memory clinic patients from the Amsterdam Dementia Cohort diagnosed with AD (*n* = 65) or MCI (*n* = 61) and those deemed cognitively normal (*n* = 37) who had CSF collected at two time points [22]. At baseline all patients underwent CSF collection and standard dementia screening, including physical and neurological examination, laboratory tests, electroencephalography, and magnetic resonance imaging. Cognitive screening included a Mini-Mental State Examination (MMSE; range 0–30, with 30 indicative of perfect performance), but usually also involved comprehensive neuropsychological testing. The diagnosis of probable AD was made according to National Institute of Neurological and Communicative Disorders and Stroke–Alzheimer’s Disease and Related Disorders Association criteria [32]. The diagnosis of MCI was made according to Petersen criteria [37], and participants met the core clinical criteria for MCI of the National Institute on Aging–Alzheimer’s Association [2]. When the results of all examinations were normal, patients were considered to have subjective memory complaints. The cognitively normal group consisted of 31 individuals with subjective memory complaints, two patients with a psychiatric disorder, two patients with temporal epilepsy, and two healthy volunteers. Diagnoses were made by consensus of a multidisciplinary team without regard to CSF biomarker results. The study was approved by the ethical review board of the VU University Medical Center, and all individuals gave written informed consent.

### Follow-up

Patients were followed-up at the Amsterdam clinic. Within the MCI group (follow-up mean (standard error (SE)) 2.7 (0.3) years; range 0.1–10.9 years) 17 patients remained stable, and 36 progressed to symptomatic AD [32] and eight to other dementias (two to fronto-temporal lobar degeneration [34], three to vascular dementia [39], one to dementia with Lewy bodies, one to progressive supranuclear palsy [31], and one diagnosed with normal pressure hydrocephalus). Within the 37 cognitively normal individuals (follow-up mean (SE) 4.0 (0.5) years; range 0.9–9.6 years; *n* = 33), six patients with subjective complaints progressed to MCI, three patients to AD, and one patient to vascular dementia, while 27 patients remained stable. During follow-up, patients were asked to undergo a second lumbar puncture (minimum interval 6 months; range 0.7–6.2 years, with a mean of 2.0 years). At baseline, VILIP-1 data were unavailable for two patients and YKL-40 data for one patient; at follow-up, VILIP-1 data were unavailable for one patient and YKL-40 data for three patients. We used all 679 available MMSE measurements to estimate annual cognitive decline over time (MMSE at follow-up was available in 31 cognitively normal individuals, 59 MCI patients, and 58 AD patients; mean (SE) 3.8 (0.2) years).

### CSF analyses

CSF was obtained by lumbar puncture using a 25-gauge needle and collected in 10 ml polypropylene tubes. Within 2 h, CSF samples were centrifuged at 1800 × *g* for 10 min at 4 °C. CSF was aliquoted in polypropylene tubes of 0.5 or 1 ml and stored at −80 °C until further analysis. Baseline CSF Aβ42, tau, and ptau-181 were measured with INNOTEST ELISA (Fujirebio Europe (formerly Innogenetics), Gent, Belgium) at the VU University Medical Center, Amsterdam [9]. The inter-assay coefficient of variation (%CV) (mean ± standard deviation) was 10.9 ± 1.8 % for Aβ42, 9.9 ± 2.1 % for tau, and 9.1 ± 1.8 % for ptau-181, as analyzed in a high pool and a low pool from 13 consecutive pool preparations used in total in 189–231 runs. At Washington University in St. Louis, CSF samples were analyzed for YKL-40 by enzyme-linked immunosorbent assay (ELISA; Quidel, San Diego, CA, USA) and for VILIP-1 by a microparticle-based immunoassay (Erenna; Singulex, Alameda CA, USA) [15, 42]. Intra-assay and inter-assay %CV values were 4.4 % and 10.6 % for YKL-40, and were 3.1 % and 8.6 % for VILIP-1, respectively. To circumvent inter-assay variability, baseline and follow-up samples were analyzed on the same assay plate [11, 46].

### Statistical analysis

Cross-sectional differences among diagnostic categories were assessed using analysis of variance (ANOVA), adjusted for sex and age, with post-hoc Bonferroni corrections, or the Fisher exact test when applicable. For ANOVA, CSF biomarkers were log-transformed to fit the assumptions needed for the model. For subgroup analyses with ANOVA, the MCI group was divided into stable MCI patients and MCI patients progressing to symptomatic AD, and analyses were performed in the same manner. Cox proportional hazard models adjusting for sex and age were performed to analyze the predictive value of CSF biomarkers for progression of MCI to AD, and then for progression of MCI to all types of dementia. For the Cox analyses we used the CSF biomarkers in tertiles (YKL-40: lowest tertile, <219 ng/ml; middle tertile, 219–328 ng/ml; highest tertile, > 328 ng/ml; VILIP-1: lowest tertile, <131 pg/ml; middle tertile, 131–200 pg/ml; highest tertile, >200 pg/ml). For all analyses, the lowest tertile was used as the reference. The same analyses were also performed using CSF biomarkers as continuous variables. Hazard ratios (HRs) are presented with a 95 % confidence interval (CI). Kaplan–Meier curves were made for illustrative purposes. In addition, we estimated the effect of baseline levels of YKL-40 and VILIP-1 on cognitive decline as defined by repeated MMSE. For this purpose, we used linear mixed models with the baseline CSF biomarker level (YKL-40 or VILIP-1) in tertiles (as already described), time (years), and interaction between time and CSF biomarker as independent variables, and the MMSE score as the dependent variable. We adjusted for age and sex, and analyses were performed separately for each diagnostic category. All available MMSE scores were taken into account, and all mixed models were specified with a random intercept and/or slope based on –2 Log Likelihood criteria [45]. Finally, age and sex-adjusted linear mixed models were applied to assess within-person annual changes over time in CSF biomarker levels in each of the diagnostic groups. The CSF biomarkers (YKL-40 and VILIP-1) were the dependent variables (each in a separate model), while diagnosis (treated as a categorical variable), time (in years; treated as a continuous variable), and interaction between diagnosis and time were independent variables. Diagnostic categories were recoded as dummy variables to be able to estimate the mean (SE) for each category. For statistical analyses, IBM SPSS 21 (for Windows; Armonk, New York, USA) was used. Statistical significance was set at *p* ≤0.05.

## Results

### Baseline characteristics

MCI patients were older than those who were cognitively normal, as shown in Table 1. MMSE scores were lower in both symptomatic AD and MCI individuals compared with cognitively normal individuals, and lower in AD compared with MCI. *APOE* ε4 carriership was more common in AD than in cognitively normal individuals. CSF Aβ42 levels were, as expected, lower in both AD and MCI groups compared with those who were cognitively normal, and lower in AD compared with MCI. CSF tau and ptau-181 levels were higher in both AD and MCI groups compared with those who were cognitively normal.

**Table 1:** Patient baseline characteristics and CSF biomarkers in diagnostic groups

	Cognitively normal	MCI	AD
	(*n* = 37)	(*n* = 61)	(*n* = 65)
Age (years)	64 (2)	68 (1)^*^	65 (1)
Sex, female	14 (38 %)	23 (38 %)	29 (45 %)
MMSE at baseline (range 0–30)^a^	28 (0.3)	27 (0.3)^*^	22 (0.7)^†,‡^
*APOE* genotype, ε4 carrier^b^	15 (42 %)	33 (57 %)	45 (70 %)^*^
Follow-up time (years)	2.4 (0.2)	2.0 (0.1)	1.9 (0.1)
CSF biomarkers			
Aβ42 (pg/ml)	741 (44)	530 (32)^†^	412 (18)^†,‡^
tau (pg/ml)	349 (38)	606 (64)^†^	688 (44)^†^
ptau-181 (pg/ml)	54 (4)	78 (6)^†^	86 (4)^†^

Data presented as mean (standard error) or number (percentage). Fisher’s exact test or ANOVA with post-hoc Bonferroni corrections were used when applicable. CSF biomarkers were log-transformed for ANOVA analyses

^a^Baseline MMSE (with 30 indicative of perfect performance) was available for 160 patients, and follow-up MMSE was available for 148 patients; the cognitive follow-up period was (mean (standard error) 3.8 (0.2) years)

^b^ *APOE* genotype data were available for 36 cognitively normal individuals, 58 MCI patients, and 64 AD patients (total 158)

^*^ *p* ≤ 0.05 vs. cognitively normal

^†^ *p* ≤ 0.005 vs. cognitively normal

^‡^ *p* ≤ 0.005 vs. MCI

*Aβ42* amyloid beta 1–42, *AD* Alzheimer’s disease, *ANOVA* analysis of variance, *CSF* cerebrospinal fluid, *MCI* mild cognitive impairment, *MMSE* Mini-Mental State Examination, *ptau-181* tau phosphorylated at threonine 181, *tau* total tau

### Levels of CSF biomarkers at baseline as a function of clinical diagnosis

Mean baseline YKL-40 levels were different among the three diagnostic groups (*p* = 0.006). Pair-wise comparisons with Bonferroni corrections showed that baseline YKL-40 levels were higher in MCI and AD patients, compared with the cognitively normal individuals (mean (SE) 304 (16) and 288 (12) ng/ml vs. 231 (16) ng/ml, *p* = 0.03 and *p* = 0.006), as shown in Table 2. Although mean baseline levels of VILIP-1 in MCI and AD patients were higher than those in cognitively normal individuals (mean (SE), 192 (13) and 182 (10) pg/ml vs. 168 (11) pg/ml), the differences were not statistically significant (effect for diagnosis *p* = 0.88).

**Table 2:** Baseline levels of CSF biomarkers and change within individuals over time

	Cognitively normal	MCI	AD
	(*n* = 37)	(*n* = 61)	(*n* = 65)
YKL-40 (ng/ml), baseline	231 (16)	304 (16)^*^	288 (12)^*^
YKL-40 (ng/ml), follow-up	241 (18)	320 (16)^*^	306 (14)^†^
Annual change, (β (SE))	5.3 (3.2)	8.9 (3.0)^‡^	7.1 (3.1)^§^
VILIP-1 (pg/ml), baseline	168 (11)	192 (13)	182 (10)
VILIP-1 (pg/ml), follow-up	175 (11)	217 (14)	190 (11)
Annual change, (β (SE))	2.8 (2.8)	10.7 (2.6)^‡^	3.1 (2.6)

Data presented as mean (SE). At baseline, VILIP-1 data were missing for two patients and YKL-40 data for one patient; at follow-up, VILIP-1 data were missing for one patient and YKL-40 data for three patients. Baseline and follow-up differences (mean (SE) LP interval was 2.0 (0.1) years) were assessed with ANOVA with post-hoc Bonferroni corrections, adjusted for age and sex. CSF biomarkers were log-transformed for ANOVA analyses. Longitudinal effects were assessed using age and sex-adjusted linear mixed models, with CSF biomarkers (VILIP-1 and YKL-40) as dependent variables and clinical diagnosis (categorical), time (LP interval in years), and interaction diagnosis × time as independent variables. The reported β value represents the estimated change of YKL-40 (ng/ml) or VILIP-1 (pg/ml) levels per year

^*^ *p* ≤0.05 vs. cognitively normal subjects

^†^ *p* ≤0.005 vs. cognitively normal subjects

^‡^ *p* ≤0.005 for time effect

^§^ *p* ≤0.05 for time effect

*AD* Alzheimer’s disease, *ANOVA* analysis of variance, *CSF* cerebrospinal fluid, *LP* lumbar puncture, *MCI* mild cognitive impairment, *SE* standard error, *VILIP-1* Visinin-like protein-1, *YKL-40* Chitinase-3-like protein 1

### Predictive value of baseline levels of biomarkers for progression of MCI to symptomatic AD

**Fig. 1:**
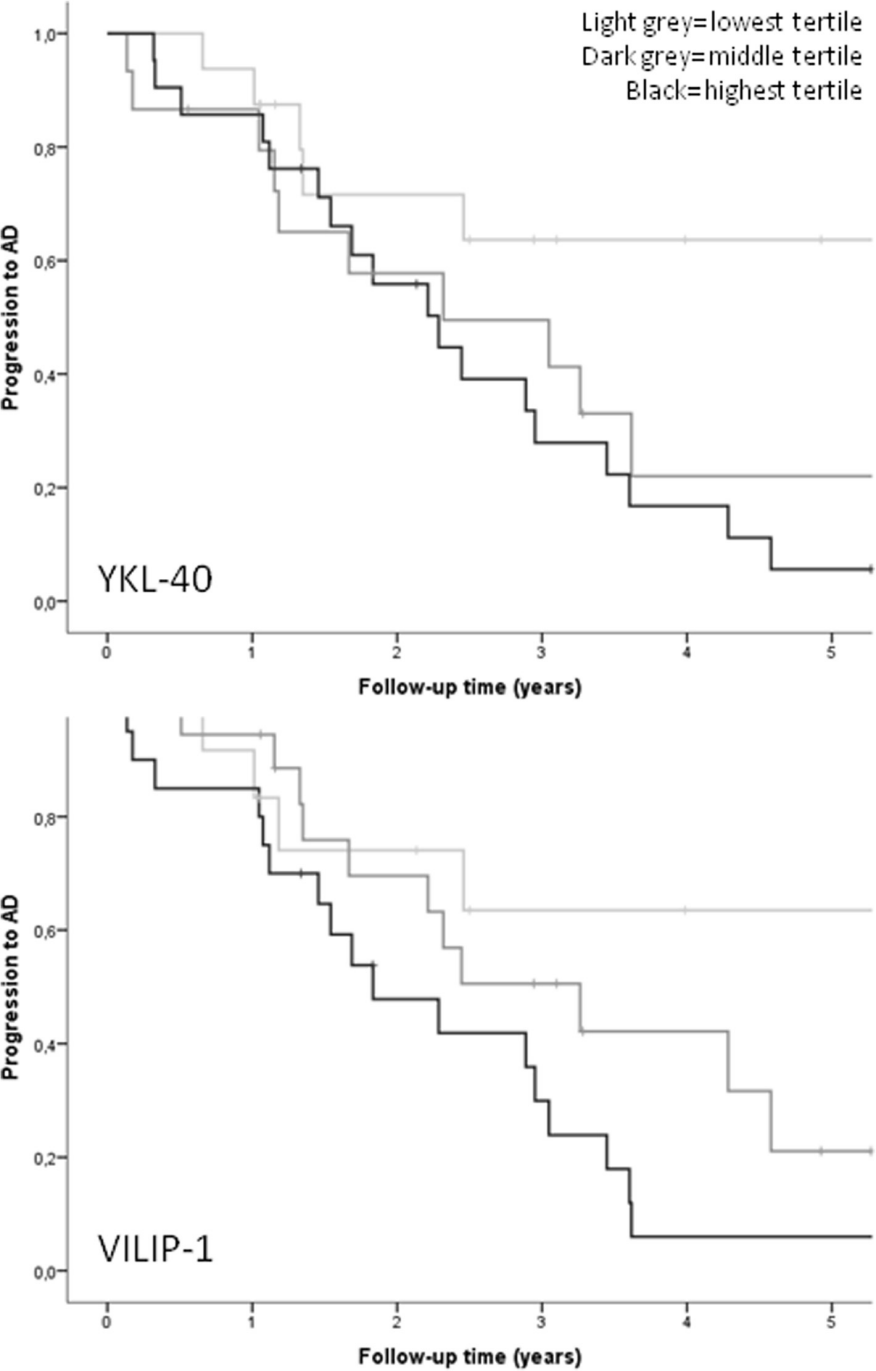
Kaplan–Meier curve for progression of MCI to AD. Progression of MCI to AD for tertiles of YKL-40 (*upper*) and VILIP-1 (*lower*). For YKL-40: lowest tertile, <219 ng/ml (*light gray line*); middle tertile, 219–328 ng/ml (*dark gray line*); and highest tertile, >328 ng/ml (*black line*). For VILIP-1: lowest tertile, <131 pg/ml (*light gray line*); middle tertile, 131–200 pg/ml (*dark gray line*); and highest tertile, >200 pg/ml (*black line*). Lowest tertile used as reference. *AD* Alzheimer’s disease, *VILIP-1* Visinin-like protein-1, *YKL-40* Chitinase-3-like protein 1

Further analyses with ANOVA showed that baseline levels of YKL-40 were higher in MCI patients who progressed to AD (*n* = 36) compared with stable MCI patients (*n* = 17, mean (SE), 327 (19) ng/ml vs. 242 (31) ng/ml, *p* = 0.01), as shown in Table 3. Baseline levels of YKL-40 were also predictive of progression from MCI to AD, with HR (95 % CI) of 3.0 (1.1–7.9) for the highest tertile and 2.9 (1.0–8.1) for the middle tertile of YKL-40, compared with the reference (lowest tertile), as shown in Fig. 1.

**Table 3:** Baseline characteristics and CSF biomarkers in mild cognitive impairment

	sMCI	MCI-AD
	(*n* = 17)	(*n* = 36)
Age (years)	64 (2)	70 (1)^*^
Sex, female	6 (35 %)	13 (36 %)
MMSE (range 0–30)^a^	28 (0.6)	26 (0.4)^*^
Aβ42	579 (493–814)	410 (322–507)^**^
tau	274 (212–418)	739 (463–950)^**^
ptau-181	47 (40–79)	90 (65–124)^**^
YKL-40 (ng/ml), baseline	242 (31)	327 (19)^*^
YKL-40 (ng/ml), follow-up	247 (23)	363 (22)^*^
Annual change, (β(SE))	10.2 (6.4)	10.1 (3.1)^‡^
VILIP-1 (pg/ml), baseline	136 (25)	233 (17) ^**^
VILIP-1 (pg/ml), follow-up	167 (20)	256 (19)^**^
Annual change, (β(SE))	16.4 (6.1)^§^	12.0 (3.0)^‡^

Data presented as mean (SE) or number (percentage). At baseline VILIP-1 data were missing for two patients and YKL-40 for one patient. Fisher’s exact test or ANOVA with post-hoc Bonferroni corrections was used when applicable. CSF biomarkers were log-transformed for ANOVA analyses. Longitudinal effects were assessed using age and sex-adjusted linear mixed models, with CSF biomarkers (VILIP-1 and YKL-40) as dependent variables and clinical diagnosis (sMCI vs. MCI-AD), time (LP interval in years), and interaction diagnosis × time as independent variables. The reported β value represents the estimated change of YKL-40 (ng/ml) or VILIP-1 (pg/ml) levels per year

^a^Baseline MMSE was available for 52 patients

^*^ *p* ≤0.05 vs. sMCI

^**^ *p* ≤0.005 vs. sMCI

^‡^ *p* ≤0.005 for time effect

^§^ *p* ≤0.05 for time effect

*Aβ42* amyloid beta 1–42, *ANOVA* analysis of variance, *CSF* cerebrospinal fluid, *LP* lumbar puncture, *MCI-AD* mild cognitive impairment progressing to Alzheimer’s disease, *MMSE* Mini-Mental State Examination, *ptau-181* tau phosphorylated at threonine 181, *sMCI* stable mild cognitive impairment, *SE* standard error, *tau* total tau, *VILIP-1* Visinin-like protein-1, *YKL-40* Chitinase-3-like protein 1

Similar to the results observed for YKL-40, ANOVA analyses showed that baseline levels of VILIP-1 were higher in MCI patients that progressed to AD (*n* = 35) compared with stable MCI patients (*n* = 17) (mean (SE) 233 (17) pg/ml vs. 136 (25) pg/ml, *p* = 0.001, respectively). Baseline levels of VILIP-1 in individuals with MCI were predictive of progression to AD, with HR (95 % CI) of 4.4 (1.5–13.0) for the highest tertile and 2.1 (0.7–6.7) for the middle tertile, compared with the reference (lowest tertile).

When repeating these analyses with CSF biomarkers as continuous variables, effects remained the same (HR (95 % CI): YKL-40 1.003 (1.001–1.006), *p* = 0.02; VILIP-1 1.005 (1.002–1.008), *p* = 0.003). In addition, results were virtually identical when analyzing for progression of MCI to all types of dementia (*n* = 44) instead of progression to AD alone (HR (95 % CI): YKL-40 3.2 (1.3–7.9) for the highest tertile and 2.9 (1.1–7.2) for the middle tertile; VILIP-1 3.8 (1.5–9.4) for the highest tertile and 1.7 (0.6–4.4) for the middle tertile).

### Predictive value of baseline levels of biomarkers for cognitive decline as measured by MMSE

Baseline levels of YKL-40 were predictive for cognitive decline in AD (β (SE) 0.65 (0.29), *p* = 0.03), but not in those who were cognitively normal or had MCI (β (SE) –0.10 (0.13), *p* = 0.44 and −0.32 (0.19), *p* = 0.11, respectively). Cognitive decline for those AD patients in the lowest tertile of YKL-40 was 2.8 MMSE points per year, while patients with levels in the highest tertile decreased 1.5 points annually. Baseline levels of VILIP-1 were predictive of cognitive decline as measured by MMSE in MCI (β (SE) –0.39 (0.19), *p* = 0.05), but not in cognitively normal or AD patients (β (SE) 0.01 (0.15), *p* = 0.95 and 0.13 (0.27), *p* = 0.64, respectively). Cognitive decline in MCI with VILIP-1 levels in the lowest tertile was 0.7 MMSE points per year, while patients with levels in the highest tertile declined 1.5 MMSE annually.

### Longitudinal changes of biomarkers

**Fig. 2:**
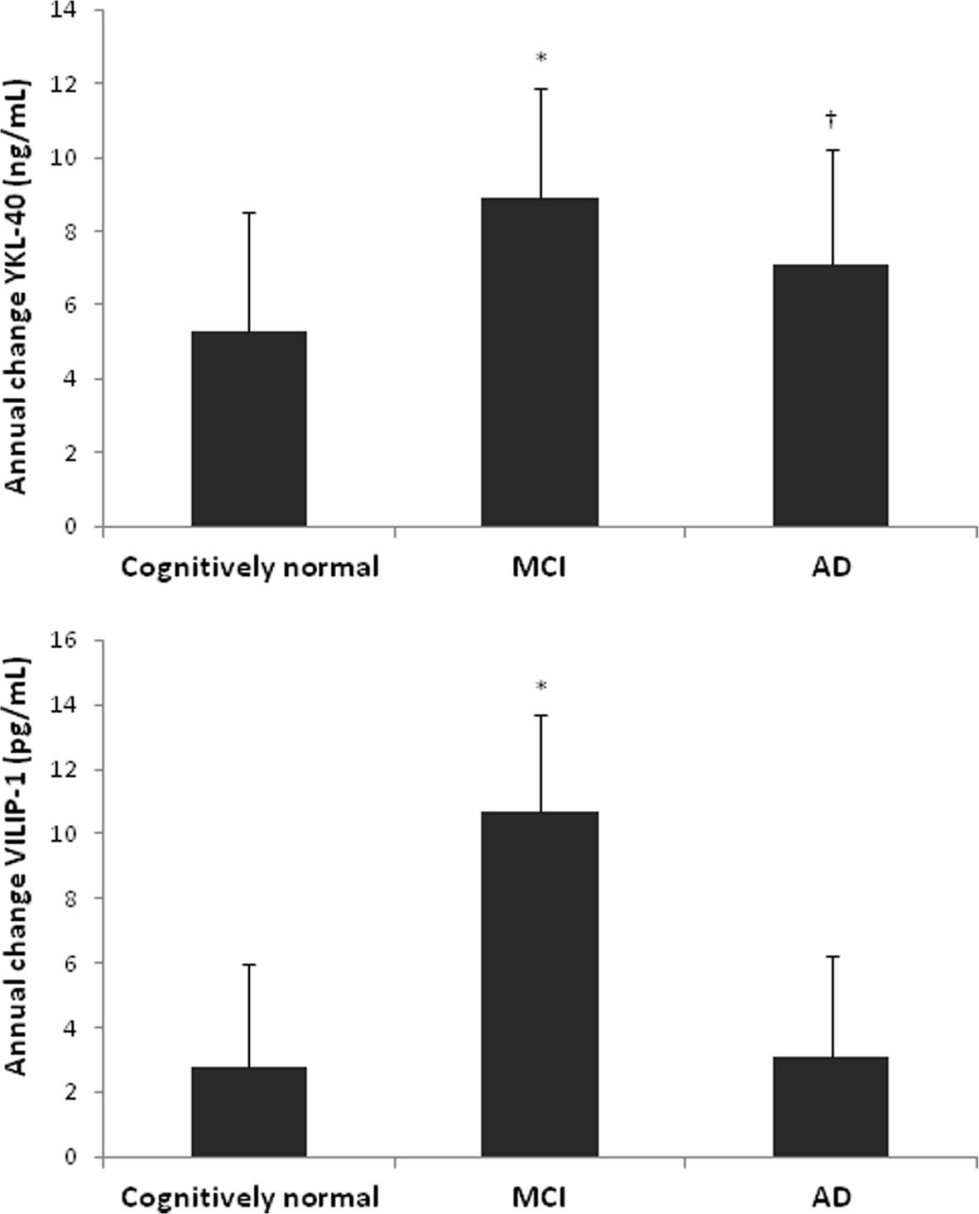
Annual increase of CSF levels of YKL-40 and VILIP-1. Annual changes of CSF biomarker levels were assessed using age and sex-adjusted linear mixed models, with CSF biomarkers (VILIP-1 and YKL-40) as dependent variables and clinical diagnosis (categorical), time (LP interval in years), and interaction diagnosis × time as independent variables. The reported β value represents the estimated change of YKL-40 (ng/ml) or VILIP-1 (pg/ml) levels per year. Error bars represent SE of the reported effect. ^*^ *p* ≤0.005 for time effect, †*p* ≤0.05 for time effect. *AD* Alzheimer’s disease, *MCI* mild cognitive impairment, *VILIP-1* Visinin-like protein-1, *YKL-40* Chitinase-3-like protein 1

Mixed model analyses showed that levels of YKL-40 within individuals increased in both MCI and AD patients (mean (SE) 8.9 (3.0) ng/ml per year, *p* = 0.004 and 7.1(3.1) ng/ml per year, *p* = 0.02, respectively), but not in the cognitively normal group (*p* = 0.10), as shown in Table 2 and Fig. 2.

Levels of VILIP-1 increased over time in MCI patients by mean (SE) 10.7 (2.6) pg/ml per year (*p* <0.001), but levels did not change significantly in cognitively normal individuals or AD patients (*p* = 0.31 and *p* = 0.23, respectively).

## Discussion

Baseline levels of both YKL-40 and VILIP-1 in CSF were higher in MCI patients who progressed to AD, compared with those who were clinically stable, and higher levels of both biomarkers predicted progression from MCI to symptomatic AD. Results for progression to all types of dementia (including but not limited to AD) were essentially the same, suggesting that these biomarkers reflect pathologies that are not specific for AD (e.g., inflammation and/or neurodegeneration). In addition, we confirm that levels of YKL-40 were higher in MCI and AD patients compared with cognitively normal individuals. Furthermore, we show that levels of YKL-40 increased over time in both MCI and AD, and that levels of VILIP-1 increased over time in MCI, indicating that both of these CSF biomarkers might be useful for disease monitoring.

The finding of higher levels of YKL-40 in MCI and AD compared with cognitively normal individuals is consistent with previous studies in CSF and brain [3, 4, 15, 26, 30, 35]. Our results also show that higher YKL-40 levels were predictive for development of subsequent cognitive impairment in MCI, a finding that is consistent with data from a large group of cognitively normal individuals in an independent cohort [15], but differs slightly from results of another study [35]. Within individuals with AD dementia, lower levels of YKL-40 were associated with a more rapid cognitive decline than were higher levels, which seems counterintuitive. Further studies evaluating a more complex set of cognitive tests will allow for a testing of the validity and implications of these results.

We found that YKL-40 levels increased longitudinally during the stages of MCI and AD, but not in cognitively normal individuals, which implies that the increment over time reflects clinical (symptomatic) disease progression. Although the role of YKL-40 in AD has not been completely elucidated, the protein appears to be expressed in astrocytes and/or microglia, with expression associated with reactive gliosis and neuroinflammation [7, 8, 15, 30]. Levels of YKL-40 in CSF are also increased following stroke, and in other neurological disorders, indicating that it is not specific for AD but instead seems to be a more general marker for inflammation [8, 19, 28, 29]. Our finding of increases in YKL-40 over time in both MCI and AD is consistent with the fact that inflammation is a key process in disease progression in AD [1] and as such could be a useful marker for monitoring disease progression.

Levels of VILIP-1 increased over time in patients with MCI, but not in those with AD dementia. VILIP-1 is a brain-specific neuronal calcium-sensor protein which may play a role in signal transduction and neurotransmission [12, 24]. It also has been associated with increased hyperphosphorylation of tau in the brain, and neuronal cell death [13, 40]. Our results suggest that the pathological processes associated with increased levels of VILIP-1, such as neuronal dysfunction and death, are mainly active in an earlier phase of the disease. This pattern is consistent with findings in individuals carrying AD-causing gene mutations [17]. In cross-sectional analyses, the present results showed higher levels of VILIP-1 in individuals with MCI and AD compared with cognitively normal individuals; however, this difference did not reach statistical significance in contrast to prior publications [17, 25, 27, 42, 43]. Of note, the absolute levels of VILIP-1 in the present study were roughly twofold lower than those reported in the previous studies that used the same assay, probably owing to the recent improvement in assay calibration. However, the relative differences between the clinical groups were the same in both sets of studies. The lack of statistical difference could reflect the relatively small number of cognitively normal subjects evaluated (*n* = 37), the fact that a fair number of controls progressed to some having MCI or dementia over 4 years (*n* = 10), and differences between the demographic and clinical characteristics of the control groups between sets of studies. The cognitively normal group in the current study was biased towards patients who showed decline (six patients progressed to MCI and four patients to dementia), and the percentage of *APOE* ε4 carriership in our cognitively normal group was higher than reported in the previous studies (i.e., 42 % vs. 17–29 %) [27, 43], and higher than in the general population (20–25 %) [38], suggesting a potentially higher prevalence of preclinical AD in this population. Such differences could have diluted the diagnosis effect. The lack of difference between our “control” and AD groups should be interpreted with caution. In our cohort we also observed a high rate of progression to AD in the MCI group. This observation may reflect a selection bias in that progressors may be more likely to return to our clinic for a second lumbar puncture. Despite these differences and limitations, we confirm that higher VILIP-1 levels are associated with progression of MCI to AD and more rapid cognitive decline in MCI [42, 43]. Furthermore, in this longitudinal cohort we were able evaluate biomarker change over time as a “biological marker” for disease progression. It will be of interest to evaluate the utility of this marker in memory clinic settings for predicting progression of cognitively normal individuals to MCI once we obtain a larger number of individuals and years of clinical follow-up. Ideally, evaluation of longitudinal changes in CSF YKL-40 and VILIP-1 will be performed in a cohort of individuals as they progress from being cognitively normal to having mild cognitive impairments and eventually AD dementia. This would permit the characterization of the true changes in these markers with disease progression.

## Conclusions

We studied two relatively novel CSF biomarkers for AD in a group of memory clinic patients, both cross-sectionally and longitudinally. Both YKL-40 and VILIP-1 were associated with clinical disease progression. VILIP-1 seems of most value in earlier stages (i.e., MCI), and could be a marker of neuronal injury and early decline, reaching a plateau earlier than YKL-40. In the setting of previously proposed models, our data suggest that YKL-40 CSF levels begin to increase before the stage of MCI and keep increasing during the symptomatic stages of AD in contrast to the more classic CSF biomarkers that reach a plateau [21, 36, 41]. Results from in vitro and animal studies suggest that YKL-40 is a nonspecific biomarker for inflammation which is a key process in AD disease progression [20]. YKL-40 could thus be especially valuable in monitoring and predicting disease progression within the phase of clinical (symptomatic) AD, possibly also in the setting of future modifying therapies in AD, especially those that would target inflammatory processes.

## Abbreviations

AD: Alzheimer’s disease
Aβ42: Amyloid beta 1–42
ANOVA: Analysis of variance
CSF: Cerebrospinal fluid
CI: Confidence interval
%CV: Coefficient of variation
ELISA: Enzyme-linked immunosorbent assay
HR: Hazard ratio
MCI: Mild cognitive impairment
MMSE: Mini-Mental State Examination
ptau-181: Tau phosphorylated at threonine 181
SE: Standard error
tau: Total tau
VILIP-1: Visinin-like protein-1
YKL-40: Chitinase-3-like protein 1
